# Vascular closure strategies in pulmonary vein isolation to reduce vascular complications and facilitate same-day discharge

**DOI:** 10.1016/j.hroo.2026.07.005

**Published:** 2026-07-13

**Authors:** Isabelle N. Bax, Marisa van der Graaf, Nina F.T. Keijzer, Marije de Lange, Jippe C. Balt, Wouter R. Berger, Vincent F. van Dijk, Gerjan de Roest, Maurits C.E.F. Wijffels, Max Liebregts, Lucas V.A. Boersma

**Affiliations:** 1 Department of Cardiology, St. Antonius Hospital, Nieuwegein, The Netherlands; 2 Department of Cardiology, Amsterdam UMC, Amsterdam, The Netherlands; 3 Department of Cardiology, Rivierenland Hospital, Tiel, The Netherlands

**Keywords:** Atrial fibrillation, Catheter ablation, Pulmonary vein isolation, Vascular closure, Vascular closure devices, Access-site bleeding, Same-day discharge

## Abstract

**Background:**

Minor access-site bleeding is common after pulmonary vein isolation (PVI) for atrial fibrillation. Vascular closure devices (VCDs) may reduce bleeding, facilitating shorter bed rest and same-day discharge (SDD).

**Objective:**

This study assessed the impact of closure strategies (Perclose™ ProStyle™ [VCD] and figure-of-8 [FO8]-suture) and mobilization protocols on access-site bleeding complications and length of hospital stay.

**Methods:**

This single-center observational study included patients undergoing a primary PVI for atrial fibrillation between January 15 and December 31, 2025, at St. Antonius Hospital, the Netherlands. 4 protocols were evaluated sequentially: cohort 1: FO8-suture + 4 hours bed rest; cohort 2: VCD + 4 hours bed rest; cohort 3: VCD + 2 hours bed rest + 2 hours seated; cohort 4: VCD + FO8-suture + 2 hours bed rest + 1 hour seated. Primary outcome was in-hospital access-site bleeding, categorized by intervention. Secondary outcomes included SDD, time to discharge, unplanned overnight stay, and emergency room visits due to groin complications.

**Results:**

A total of 738 patients were included (cohorts 1–4: n = 170, 161, 210, and 197, respectively). Baseline characteristics were comparable. 2 major bleeding complications occurred in cohort 1. Minor bleeding complications decreased across the 4 cohorts from 19.4% to 9.6% (*P* = .027). Female sex (odds ratio [OR], 1.52), protamine use (OR, 0.50), and cohort 4 protocol (OR, 0.38) predicted bleeding. SDD increased from 56.5% (cohort 1) to 67.0% (cohort 4, *P* = .168), without more unplanned overnight stays or bleeding-related emergency room visits.

**Conclusion:**

The use of a VCD in combination with an FO8 suture and protamine was significantly associated with lower observed access-site bleeding complications and optimized the workflow of PVI.

**Key Findings**

▪ A vascular closure device combined with a figure-of-8 suture significantly reduced minor access-site bleeding after pulmonary vein isolation.
▪ The optimized closure strategy enabled earlier mobilization without increasing unplanned overnight stays or bleeding-related emergency department visits.
▪ Female sex was associated with a higher risk of access-site bleeding, whereas protamine use was associated with a lower risk.
▪ Optimizing vascular closure and post-procedural mobilization may improve the efficiency and safety of pulmonary vein isolation and support same-day discharge pathways.

## Introduction

Pulmonary vein isolation (PVI) by catheter ablation is a widely used treatment option for atrial fibrillation (AF) and has undergone significant advancements in recent years, resulting in shorter procedural times and improved procedural efficiency.^1^^,^^2^ With these developments and the increasing interest in striving for same-day discharge (SDD) in all patients, further optimization of the peri-procedural workflow is essential. The reported incidence of serious adverse events after PVI is low. However, vascular complications, including groin hematomas, arteriovenous fistula, and femoral pseudoaneurysms, are still commonly observed, with incidences ranging from 1% to 3%.^3^^,^^4^ Additionally, minor vascular access complications, such as small groin hematomas or persistent oozing, are even more frequently encountered after PVI,^5^^,^^6^ especially with single-shot techniques, where large-bore sheaths are used. These minor access-site bleeding events can delay ambulation and prolong post-procedural bed rest, which may subsequently lead to a prolonged hospital stay and limit the feasibility of SDD.^4^^,^^7^^,^^8^

To minimize these access-site-related complications, different options for vascular closure can be applied, including manual compression, a compression bandage, a temporary figure-of-8 (FO8) suture, the use of vascular closure devices (VCDs), and standard use of heparin antagonists. After the ablation procedure, patients must adhere to a bed-rest protocol, which may vary in duration depending on local protocol. Shorter bed-rest protocols may reduce overall hospitalization time and facilitate SDD.^4^^,^^8^, ^9^, ^10^ However, it is essential to ensure that a reduction in bed-rest duration does not lead to an increase in access-site bleeding complications.^7^ Previous studies^6^^,^^11^^,^^12^ have demonstrated that the FO8-suture is a simple and effective closure technique, although its use generally still requires many hours of post-procedural bed rest. The use of a VCD has been shown to facilitate earlier ambulation and shorten time to discharge (TTD) after AF ablation.^13^, ^14^, ^15^, ^16^ However, most studies focus on device performance in controlled settings, whereas evidence on real-world protocol-based implementation strategies and their impact on clinical workflow and access-site complications remains limited.^8^^,^^10^^,^^13^^,^^17^, ^18^, ^19^, ^20^, ^21^ Consequently, the optimal approach to minimize in-hospital access-site bleeding while simultaneously reducing post-procedural bed rest and hospitalization duration remains uncertain. Accordingly, in this study we evaluated the impact of stepwise implemented vascular closure and mobilization protocols on in-hospital access-site bleeding complications across 4 consecutive time periods in patients undergoing PVI.

## Method

### Study design

We performed a single-center observational cohort study of patients undergoing primary PVI between January 15, 2025, and December 31, 2025, at the St. Antonius Hospital, the Netherlands. This study was conducted in accordance with the Declaration of Helsinki. The local ethics committee reviewed the study as part of the Catheter Ablation for Treatment of Cardiac Arrhythmia Registry (CATCAR; Z23.067) and determined that it did not fall under the Dutch Medical Research Involving Human Subjects Act (WMO). All study data were retrospectively collected from electronic patient records, and written informed consent was not required.

### Study population

All consecutive patients undergoing a primary PVI during the study period were included. No restrictions were applied regarding the ablation modality used or other (pre-)procedural characteristics.

### Peri- and post-procedural description

During the study period, consecutive closure strategies were evaluated, introducing the implementation of a VCD (Perclose™ ProStyle™, Abbott Vascular, USA) and modifications to the post-procedural bed-rest protocol.

All procedures were performed under either general anesthesia or procedural sedation and analgesia (PSA), in accordance with the requirements of the catheter ablation system used. Both vitamin K antagonists and direct oral anticoagulants were uninterrupted before the procedure according to protocol. Vascular access was obtained with or without an ultrasound-guided puncture of the femoral vein at operator’s discretion, after which 2 sheaths were introduced: a 7 Fr sheath for a diagnostic coronary sinus catheter and a 14- 17 Fr sheath (outer diameter) for the ablation catheter. When a VCD was used, it was pre-deployed using a pre-close technique before insertion of sheaths >7 Fr, according to the institutional protocol. After positioning the diagnostic catheter into the coronary sinus, a transseptal puncture was performed under fluoroscopic guidance to access the left atrium (LA). Before insertion of the ablation catheter into the LA, patients received weight-based heparin administration (5000–10.000 IU); no activated clotting time (ACT) measurements were performed. During the study, different ablation catheters were used, including the Cryoballoon (CB; Arctic-Front Advance; Medtronic, Minneapolis, MN), outer sheath size 15.0 Fr; the pentaspline PFA catheter system (FARAWAVE™, Boston Scientific, Marlborough, MA, USA), outer sheath size 16.8 Fr; the circular over-the-wire PFA catheter system (PulseSelect™, Medtronic, Minneapolis, MN), outer sheath size 14.0 Fr and the balloon-in-basket PFA catheter system (VOLT™, Abbott, Chicago, IL), outer sheath size 17.2 Fr. Applications were delivered according to the instructions for use. At the end of the procedure, the venous access site was closed by the deployment of the previously inserted VCD and/or an FO8-suture, in addition to manual compression and application of a pressure bandage. Protamine use was at the operator’s discretion. The FO8-suture and pressure bandage were removed after 2 or 4 hours, depending on the bed-rest protocol. The following cohorts of femoral access-site closure strategies and bed-rest protocol were defined: cohort 1 (January 15–April 3, 2025): FO8-suture followed by 4 hours of post-procedural bed rest; cohort 2 (April 4–June 15, 2025): VCD for the large sheath, followed by 4 hours of post-procedural bed rest; cohort 3 (June 16–October 7, 2025): VCD for the large sheath, with post-procedural bed rest consisting of 2 hours, followed by 2 hours in a seated position before mobilization; and cohort 4 (October 8–December 31, 2025): VCD for the large sheath, combined with an FO8-suture, with post-procedural bed rest consisting of 2 hours, followed by 1 hour in a seated position before mobilization.

### Primary and secondary outcomes

The primary outcome was defined as the incidence of in-hospital access-site bleeding complications. Access-site bleeding events were classified according to the required interventions, based on the Bleeding Academic Research Consortium classification^22^ as follows: minor bleeding type 1: bleeding managed conservatively (eg, local pressure, pressure bandage, prolonged bed rest of <4 hours); minor bleeding type 2: bleeding requiring additional evaluation or therapy compared to type 1 (eg, ultrasound, prolonged bed rest of ≥4 hours, unplanned overnight stay); major bleeding type 1 (in addition to above): bleeding requiring surgical or endovascular intervention; and major bleeding type 2 (in addition to above): hemoglobin drop of >2 g/dL and/or requiring blood transfusion.

All bleeding events were initially clinically identified and scored during routine care by the treating nurse practitioner, after which the events were adjudicated by 2 independent researchers. In case of disagreement, the researchers discussed the event and reached a consensus on the bleeding classification or consulted a third party in case of disagreement.

Secondary outcomes included workflow-related outcomes: the number of patients with SDD, defined as discharge on the same day as the procedure; post-procedural length of stay, measured in hours from the end of the procedure to hospital discharge; and prolonged bed rest, including the underlying reason categorized as access-site bleeding, sedation-related, or logistic factors. Prolonged bed rest was defined as bed rest exceeding the predefined bed rest protocol mobilization. Secondary safety outcomes included unplanned overnight stay (ONS) due to bleeding and emergency room (ER) visits due to bleeding complications within 30 days after PVI.

### Statistical analysis

Patients were analyzed according to the initial treatment strategy (intention-to-treat–like approach), and remained in their originally assigned group regardless of subsequent treatment deviations, including crossover to a VCD or an FO8-suture. Continuous variables were presented as mean ± standard deviation or median with interquartile range depending on their distribution. Categorical variables were shown as absolute numbers and percentages. Normality was assessed visually using histograms and Q–Q plots.

Comparisons between categorical variables were performed using the χ^2^ test or Fisher’s exact test, as appropriate. Normally distributed continuous variables were compared using the independent samples *t*-test for 2-group comparisons or the 1-way analysis of variance for ≥3 groups. For non-normally distributed data, the Mann–Whitney U test (2 groups) or the Kruskal–Wallis test (≥3 groups) was applied. The association between study cohort and bleeding complications was expressed as odds ratios (ORs) using cohort 1 as the reference cohort.

Univariable and multivariable logistic regression analyses were performed to identify factors associated with bleeding complications. The following covariates were evaluated: body mass index (BMI), sex, ultrasound-guided femoral access, ablation catheter type (differing in Fr size), protamine administration, LA dwell time, baseline antiplatelet therapy, and study cohort (representing temporal phases of protocol implementation). Variables with a *P*-value o <.10 in the univariable analyses were entered into the multivariable logistic regression model. ORs with 95% confidence intervals (CI) were reported. Model discrimination was assessed using the area under the receiver operating characteristic curve.

Additional regression analyses were performed using cohort 4 as the reference group to compare bleeding outcomes between cohort 4 and the earlier cohorts after adjustment for the same variables as described above. Moreover, the same analysis was performed excluding patients receiving protamine to assess the independent association between closure strategy and bleeding outcomes.

To identify factors associated with TTD, univariable and multivariable linear regression analyses were performed among patients with planned SDD. The following covariates were evaluated: age, body mass index, sex, CHA_2_DS_2_-VA score, type of anesthesia, ultrasound-guided femoral access, ablation catheter type (differing in Fr size), study cohort (representing temporal phases of protocol implementation), procedural duration, and in-hospital access-site bleeding. Variables with a *P*-value of <.10 in the univariable analyses were entered into the multivariable linear regression model. Regression coefficients (β) with 95% CI were reported.

Statistical significance was defined as a 2-sided *P*-value of <.05. All analyses and visualizations were performed using R statistical software (version 4.4.1; R Foundation for Statistical Computing, Vienna, Austria).

## Results

Between January 15, 2025, and December 31, 2025, a total of 738 patients were included in this study, comprising a total of 170 patients in cohort 1, 161 patients in cohort 2, 210 patients in cohort 3, and 197 patients in cohort 4. Baseline characteristics were stratified according to cohort and shown in Table 1. The mean age was 63.6 ± 9.4 years; females comprised 36% (n = 264) of the total cohort. None of the baseline characteristics showed significant differences between treatment cohorts. All patients were analyzed according to the initial treatment strategy (intention-to-treat–like approach) and remained in their originally assigned groups. Protocol deviations, including their frequency and indications, are described in Supplemental Appendix 1.

**Table 1:** Baseline characteristics

Variable	Cohort 1 (N = 170)	Cohort 12 (N = 161)	Cohort 3 (N = 210)	Cohort 4 (N = 197)	*P*-value
Female gender (%)	58 (34.1)	51 (31.7)	81 (38.8)	74 (37.6)	.482
Age, years	63.2 ± 8.9	63.2 ± 8.8	64.0 ± 10.3	63.7 ± 9.2	.825
Body mass index, kg/m2	26.7 [23.9, 29.5]	27.1 [25.0, 29.4]	27.0 [24.2, 29.8]	26.6 [24.5, 29.4]	.718
Hypertension (%)	68 (40.0)	60 (37.3)	86 (41.0)	85 (43.1)	.728
Diabetes mellitus (%)	14 (8.2)	14 (8.7)	19 (9.0)	21 (10.7)	.862
Vascular disease (%)	19 (11.2)	13 (8.1)	24 (11.4)	24 (12.2)	.629
Creatinine, μmol/L	82.0 [71.0, 95.8]	82.0 [70.0, 93.0]	83.0 [72.8, 95.0]	80.0 [70.0, 93.0]	.506
Chronic kidney disease (%)	5 (2.9)	4 (2.5)	8 (3.8)	2 (1.0)	.350
Paroxysmal AF (%)	111 (65.3)	113 (70.2)	137 (65.2)	149 (75.6)	.084
LAVI, mL/m^2^	30.0 [24.5, 37.0]	32.5 [26.0, 38.8]	33.0 [26.5, 39.8]	33.0 [26.7, 38.6]	.062
LVEF, %	54.9 ± 7.3	54.6 ± 7.1	53.7 ± 7.7	54.3 ± 7.1	.449
Oral anticoagulation (%)	170 (100.0)	160 (99.4)	210 (100.0)	197 (100.0)	.309
DOACs (%)	160 (94.1)	155 (96.3)	203 (96.7)	188 (95.4)	
Vitamin K antagonist (%)	10 (5.9)	5 (3.1)	7 (3.3)	9 (4.6)	
CHA_2_DS_2_-VA-score (%)					.225
0	52 (30.8)	56 (35.0)	52 (25.0)	51 (26.0)	
1	54 (32.0)	39 (24.4)	62 (29.8)	66 (33.7)	
2+	63 (37.3)	65 (40.6)	94 (45.2)	79 (40.3)	
EHRA-score (%)					.416
I	2 (1.4)	2 (1.6)	5 (2.8)	2 (1.3)	
II	116 (81.1)	90 (72.0)	141 (77.9)	117 (75.0)	
III	25 (17.5)	31 (24.8)	33 (18.2)	37 (23.7)	
IV	0 (0.0)	2 (1.6)	2 (1.1)	0 (0.0)	

Values are displayed as counts, n (%), mean ± standard deviation (SD) or median [interquartile range] as appropriate. *P*-values represent overall comparisons across study cohorts (cohorts 1–4), reflecting temporal changes during protocol implementation. AF = atrial fibrillation; DOAC = direct oral anticoagulation; EHRA = European Heart Rhythm Association; LA = left atrium; LAVI = left atrial volume index; LVEF = left ventricular ejection fraction.

### Procedural characteristics

Procedural characteristics are shown in Table 2. Ultrasound-guided femoral access significantly increased across study cohorts (from 70.6% in cohort 1 to 81.2% in cohort 4, *P* < .001), as did protamine administration after the procedure (cohort 1: 31.2% to cohort 4: 47.2%, *P* < .001). Additionally, the ablation catheter use changed during the study period, with an increased use of the balloon-in-basket PFA catheter (17.2 Fr OD) over time and reduced use of the circular over-the-wire PFA catheter (14.0 Fr OD) and cryoballoon catheter (15.0 Fr OD). LA dwell time increased from 25.0 min (22.0,33.0) in cohort 1 to 28.5 min (24.0, 36.0) in cohort 4 (*P* < .001).

**Table 2:** Procedural characteristics

Variable	Cohort 1 (N = 170)	Cohort 2 (N = 161)	Cohort 3 (N = 210)	Cohort 4 (N = 197)	*P*-value
Ablation catheter type					<.001
Pentaspline PFA catheter (16.8 Fr) (%)	88 (51.8)	71 (44.1)	88 (41.9)	133 (67.5)	
Circular over-the-wire PFA catheter (14.0 Fr) (%)	66 (38.8)	59 (36.6)	64 (30.5)	19 (9.6)	
Balloon-in-basket PFA catheter (17.2 Fr) (%)	0 (0.0)	6 (3.7)	24 (11.4)	38 (19.3)	
Cryoballoon catheter (15.0 Fr) (%)	16 (9.4)	25 (15.5)	34 (16.2)	7 (3.6)	
Ablation size outer diameter, Fr	16.8 [14.0, 16.8]	15.0 [14.0, 16.8]	16.8 [14.0, 16.8]	16.8 [16.8, 16.8]	<.001
General anesthesia (%)	139 (81.8)	127 (78.9)	160 (76.2)	156 (79.2)	.621
Ultrasound-guided punctures (%)	120 (70.6)	145 (90.1)	187 (89.0)	160 (81.2)	<.001
Procedure duration, min	25.0 [22.0, 33.0]	27.0 [23.0, 38.0]	31.0 [25.0, 41.2]	28.5 [24.0, 36.0]	<.001
Fluoroscopy duration, min	9.0 [8.0, 12.0]	10.0 [8.0, 13.0]	11.0 [9.0, 14.0]	10.0 [8.0, 13.0]	.002
Protamine use (%)	53 (31.2)	38 (23.6)	94 (45.0)	93 (47.2)	<.001
Use of figure-of-eight (%)	167 (98.8)	13 (8.1)	19 (9.0)	197 (100.0)	<.001
Use of a VCD (%)	1 (0.6)	159 (98.8)	210 (100.0)	197 (100.0)	<.001
1× VCD (%)	0 (0.0)	153 (96.2)	194 (92.4)	191 (97.0)	
2× VCD (%)	1 (100.0)	1 (0.6)	0 (0.0)	3 (1.5)	
VCD failure (%)	0 (0.0)	5 (3.1)	16 (7.6)	3 (1.5)	

Values are displayed as counts, n (%), mean ± SD, or median [interquartile range] as appropriate. *P*-values represent overall comparisons across study cohorts (cohorts 1–4), reflecting temporal changes during protocol implementation. The French size relates to the outer sheath diameter. Fr = French size; PFA = pulsed field ablation; VCD = vascular closure device.

### Primary safety outcome

Major and minor bleeding complications occurred in 16.3% of all patients. Bleeding complications included 2 major vascular complications in cohort 1, whereas no major bleeding complications were reported in other cohorts. 1 event was classified as a type 1 major bleeding, occurring despite ultrasound-guided access, due to active bleeding from the arteria circumflexa femoris medialis, which required embolization. The second event was a type 2 major bleeding, which occurred without ultrasound guidance and was characterized by a hemoglobin drop of >2 g/dL, conservatively managed with short-term hospitalization and subsequent recovery. There were 35 bleeding events (20.6%) in cohort 1, 32 (19.9%) in cohort 2, 34 (16.2%) in cohort 3, and 19 (9.6%) in cohort 4 (*P* = .017). Minor bleeding complications significantly decreased from 19.4% in cohort 1 to 9.6% in cohort 4 (*P* = .027) despite shorter bed-rest time. Distribution between minor bleeding types is shown in Table 3. Time to bleeding complications significantly decreased from a median of 271 min in cohort 1 to 157 min in cohort 4 (*P* < .001), in line with the protocol-based shortening of ambulation time by 120 minutes. Additional information on bleeding outcomes is shown in Table 3 and Figure 1.

**Table 3:** Bleeding and hospitalization outcomes

Variable	Cohort 1 (N = 170)	Cohort 2 (N = 161)	Cohort 3 (N = 210)	Cohort 4 (N = 197)	*P*-value
Access-site bleeding (%)	35 (20.6)	32 (19.9)	34 (16.2)	19 (9.6)	.017
Major type 1 (%)	1 (0.6)	0 (0.0)	0 (0.0)	0 (0.0)	.341
Major type 2 (%)	1 (0.6)	0 (0.0)	0 (0.0)	0 (0.0)	.341
Minor access-site bleeding (%)	33 (19.4)	32 (19.9)	34 (16.2)	19 (9.6)	.027
Minor type 1 (%)	20 (11.8)	21 (13.0)	26 (12.4)	17 (8.6)	.541
Minor type 2 (%)	13 (7.6)	11 (6.8)	8 (3.8)	2 (1.0)	.009
Time to first bleeding, min	271.0 [242.0, 294.2]	182.0 [148.0, 244.2]	144.5 [120.0, 185.0]	156.5 [127.5, 238.0]	<.001
Time to discharge (hours)	7.6 [5.3, 20.4]	6.2 [5.0, 19.7]	5.7 [5.0, 19.0]	5.0 [4.2, 18.9]	<.001
Time to discharge of planned SDD patients (hours)	5.4 [5.1, 6.3]	5.2 [4.8, 6.1]	5.1 [4.7, 5.7]	4.5 [4.0, 5.2]	<.001
Actual SDD	96 (56.5)	95 (59.0)	133 (63.3)	132 (67.0)	.168
Prolonged bed rest (%)	34 (20.0)	29 (18.0)	83 (39.5)	47 (23.9)	<.001
Prolonged bed rest due to bleeding (%)	31 (18.2)	26 (16.1)	34 (16.2)	18 (9.1)	.067
Prolonged bed rest due to sedation (%)	2 (1.2)	0 (0)	21 (10)	20 (10.2)	<.001
ER visit due to bleeding (%)	15 (8.8)	11 (6.8)	13 (6.2)	7 (3.6)	.215
Unplanned ONS due to bleeding (%)	4 (2.4)	2 (1.2)	5 (2.4)	2 (1.0)	.638

Values are displayed as counts, n (%), mean ± SD, or median [interquartile range] as appropriate. *P*-values represent overall comparisons across study cohorts (cohorts 1–4), reflecting temporal changes during protocol implementation. ER = emergency room; ONS = overnight stay; SDD = same day discharge.

**Figure 1:**
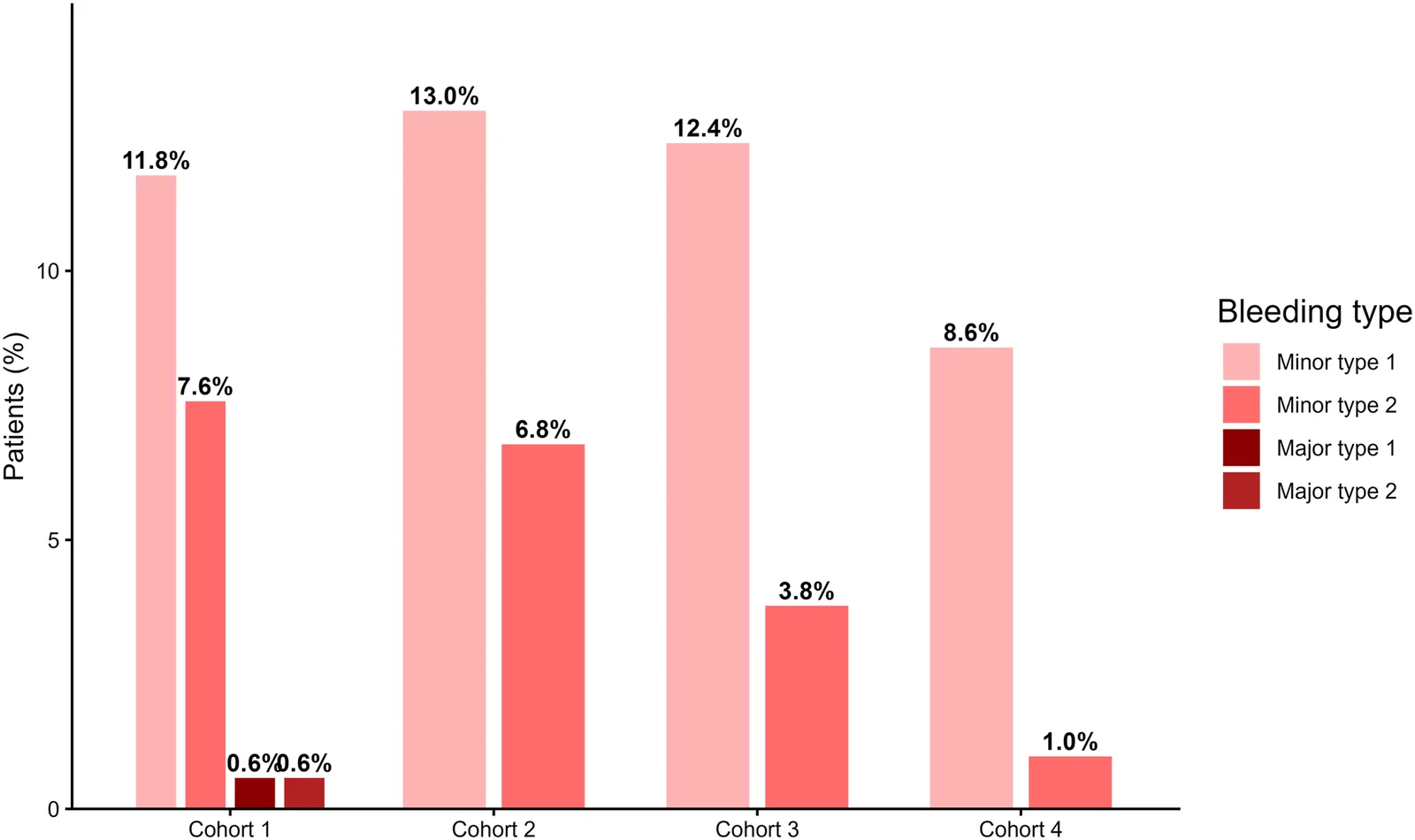
Bleeding outcomes per cohort.

### Predictors of bleeding complications

Univariable logistic regression was performed to identify predictors of bleeding complications. In the subsequent multivariable model, female sex was associated with an increased risk of bleeding (OR, 1.52; 95% CI, 1.01–2.29, *P* = .043), whereas cohort 3 (OR, 0.38; 95% CI, 0.19–0.71, *P* = .003) and protamine administration (OR, 0.50; 95% CI, 0.31–0.80, *P* = .005) were associated with reduced bleeding risk. Other variables, including ablation catheter type and sheath diameter, as well as ultrasound-guided puncture, were not significantly associated (Table 4, Figure 2A). The model showed only modest discrimination (area under the receiver operating characteristic curve, 0.65), suggesting that other factors not captured in the current analysis may have contributed to access-site bleeding risk.

**Table 4:** Univariate and multivariable logistic regression for the identification of predictors for in-hospital access-site bleeding complications

Variable	Univariable logistic regression				Multivariable logistic regression	
	OR	95% CI	*P*-value	OR	95% CI	*P*-value
Female sex	1.43	(0.96–2.13)	.078	1.52	(1.01–2.29)	**.043**
BMI	1.00	(0.95–1.05)	.929			
Antiplatelet therapy	2.08	(0.3–9.79)	.384			
Ablation catheter type						
Cryoballoon catheter (15.0 Fr)	REF					
Pentaspline PFA catheter (16.8 Fr)	0.72	(0.4–1.32)	.265	1.07	(0.58–2.04)	.838
Circular over-the-wire PFA catheter (14.0 Fr)	0.49	(0.26–0.97)	.039	0.57	(0.29–1.14)	.107
Balloon-in-basket PFA catheter (17.2 Fr)	0.59	(0.25–1.37)	.231	1	(0.4–2.43)	.999
Ablation catheter size, Fr	1.07	(0.92–1.26)	.360			
Ultrasound-guided access	0.96	(0.58–1.65)	.885			
Protamine use	0.48	(0.3–0.74)	.001	0.5	(0.31–0.8)	**.005**
Procedure time	1.01	(0.99–1.02)	.311			
Study cohort						
Cohort 1	REF					
Cohort 2	0.96	(0.56–1.64)	.872	0.9	(0.52–1.56)	.705
Cohort 3	0.73	(0.43–1.24)	.244	0.73	(0.42–1.27)	.272
Cohort 4	0.42	(0.22–0.75)	.004	0.38	(0.19–0.71)	**.003**

*P*-values represent overall comparisons across study cohorts (cohorts 1–4), reflecting temporal changes during protocol implementation. The French size relates to the outer sheath diameter. BMI = body mass index; CI = confidence interval; Fr = French size; OR = odds ratio; PFA = pulsed field ablation.

**Figure 2:**
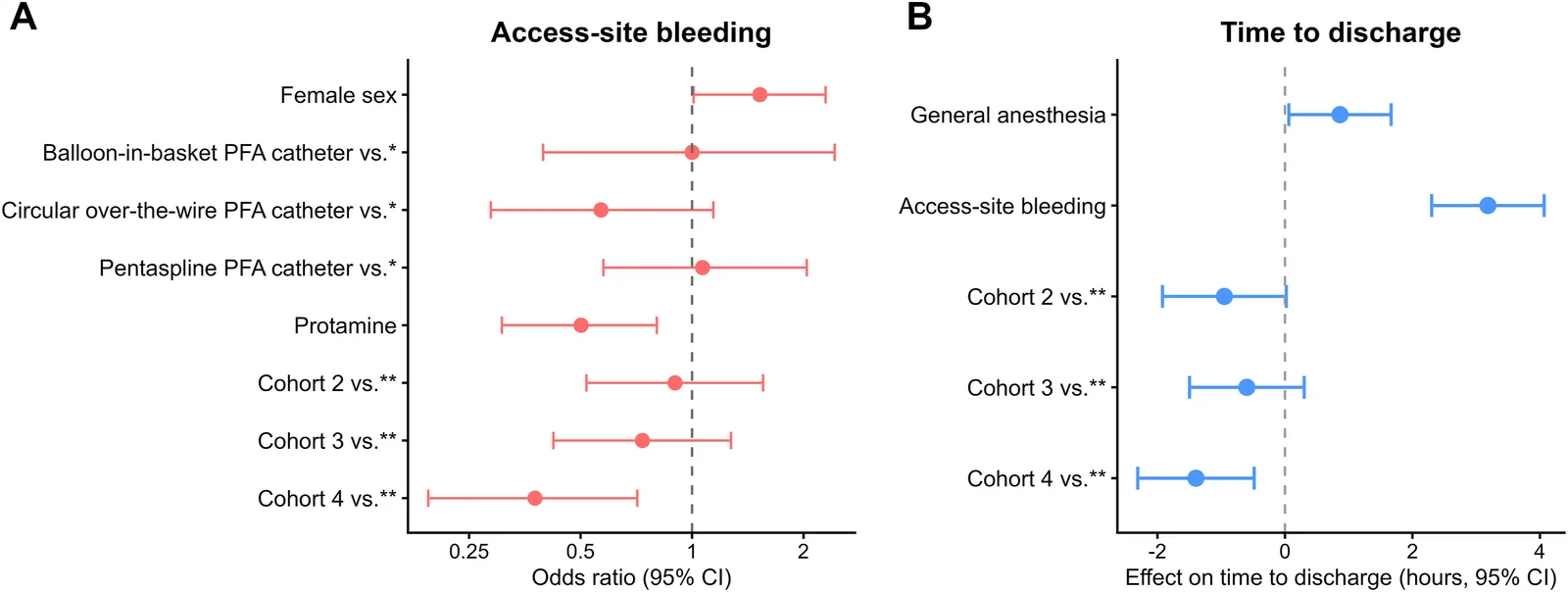
**Predictors ofaccess-sitebleeding complications and time to discharge.** (A) Forest plot of the multivariable logistic regression model identifying predictors of access-site bleeding complications. Odds ratios (ORs) with 95% confidence intervals (CIs) are shown. (B) Forest plot of the multivariable linear regression model identifying predictors of time to discharge. Regression coefficients representing the estimated effect on time to discharge (hours) with 95% CIs are shown. ∗Cryoballoon. †Cohort 1. CI = confidence interval; PFA = pulsed field ablation.

In the adjusted regression analysis using cohort 4 as the reference category, the other cohorts remained associated with significantly higher odds of bleeding, 2.02 for cohort 1 (*P* = .04), 2.26 for cohort 2 (*P* = .02), and 1.98 for cohort 3 (*P* = .04), after adjustment for procedural variables.

Additional regression analyses excluding patients receiving protamine demonstrated a similar directional association between cohort 4 and lower bleeding rates, although this no longer reached statistical significance (cohort 1 OR, 1.28; *P* = .56, and cohort 2, OR, 1.89, *P* = .13; cohort 3 OR, 1.58, *P* = .28) because patient and event numbers per group became too low to achieve appropriate statistical power.

### Secondary outcomes: Workflow related

The SDD rate in cohort 1 was 56.5%, increasing to 67.0% in cohort 4 (*P* = .168). Among patients planned for SDD, median TTD decreased from 5.4 hours in cohort 1 (5.1, 6.3) to 4.5 hours in cohort 4 (4.0, 5.2; *P* < .001). The proportion of patients requiring prolonged bed rest fluctuated between the cohorts. Although the number of patients receiving prolonged bed rest due to bleeding complications decreased by half (18.2% in cohort 1 to 9.1% in cohort 4, *P* = .067), the number of patients receiving prolonged bed rest due to post-procedural drowsiness resulting from sedation, including both general anesthesia and PSA, increased from 1.2% to 10.2% (*P* < .001) (Table 3, Figure 3B and 3C).

**Figure 3:**
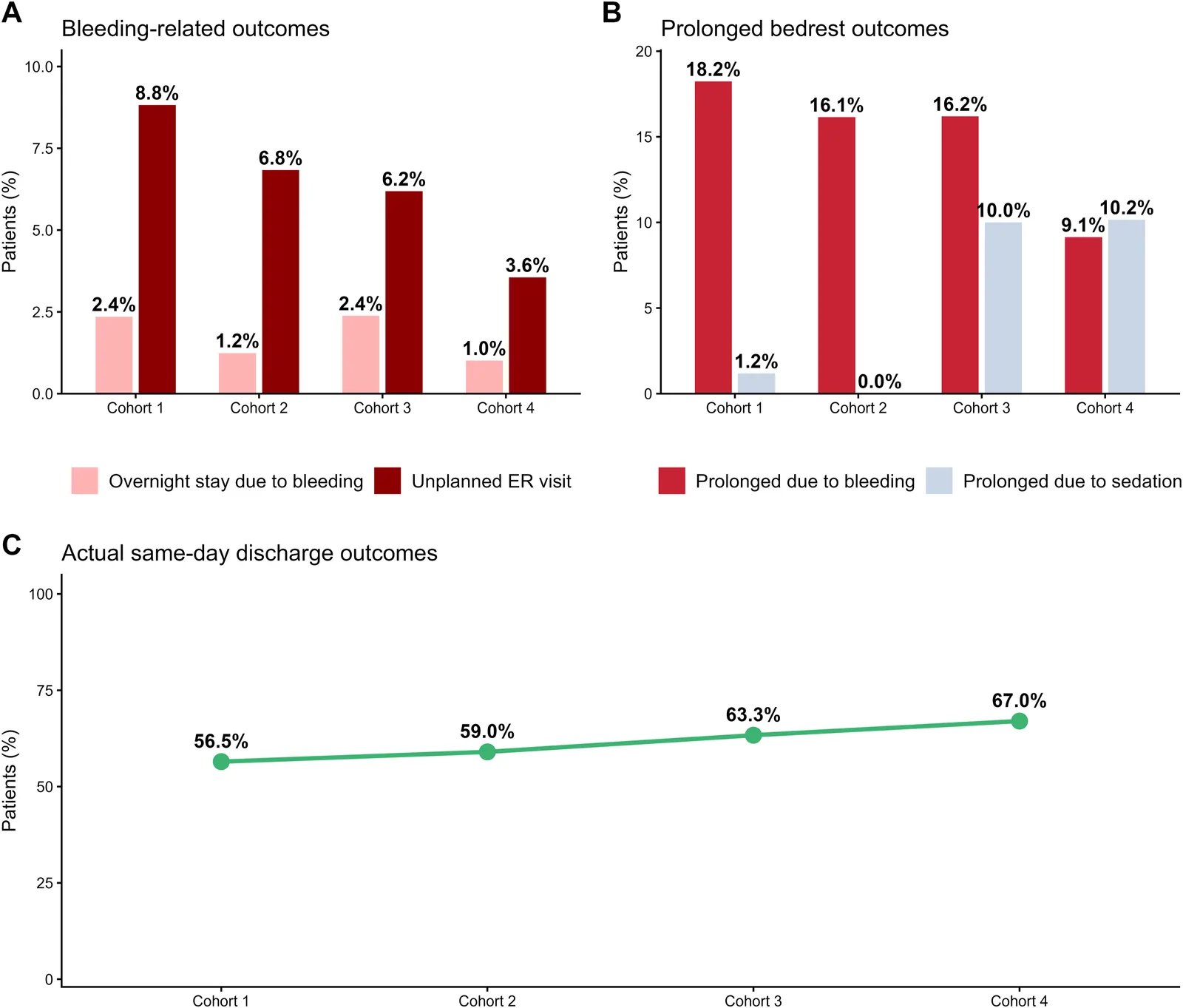
Post-procedural outcomes.

### Secondary outcomes related to bleeding events

The number of patients in need of an unplanned ONS due to bleeding, as well as ER visits related to access-site complications, showed a non-significant decrease over time (2.4% to 1.0%, *P* = .638, and 8.8% to 3.6%, *P* = .215), respectively (Figure 3A).

### Predictors of TDD

Among patients with planned SDD (n = 410), univariable linear regression identified anesthesia strategy, study cohort, and occurrence of any bleeding as potential predictors of TTD (*P* < .10). In the multivariable model, occurrence of any bleeding remained strongly associated with longer TTD (β = 3.18 hours; 95% CI, 2.30–4.07; *P* < .001). General anesthesia was also associated with prolonged discharge time (β = 0.86 hours; 95% CI, 0.06–1.67; *P* = .035), whereas cohort 4 was associated with shorter discharge time compared with cohort 1 (β = −1.40 hours; 95% CI, −2.31 to −0.48; *P* = .003) (Table 5, Figure 3). The model predicted a modest proportion of the variability in discharge time (adjusted R^2^ = 0.13).

**Table 5:** Univariate and multivariable linear regression for the identification of predictors for time to discharge in patients planned for SDD

Variable	Univariable			Multivariable linear regression		
	β	95% CI	*P*-value	β	95% CI	*P*-value
Female sex	0.29	(−0.43–1.01)	.43			
Age, years	−0.01	(−0.05–0.03)	.651			
BMI	−0.03	(−0.13–0.06)	.484			
CHA_2_DS_2_-VA-score (%)	−0.12	(−0.38–0.14)	.355			
Ablation catheter type						
Cryoballoon catheter (15.0 Fr)	REF					
Pentaspline PFA catheter (16.8 Fr)	0.70	(−0.4–1.8)	.212			
Circular over-the-wire PFA catheter (14.0 Fr)	0.70	(−0.56–1.96)	.275			
Balloon-in-basket PFA catheter (17.2 Fr)	0.18	(−1.33–1.69)	.818			
General anesthesia	0.83	(−0.03–1.69)	.058	0.86	(0.06–1.67)	**.035**
Ultrasound-guided access	0.39	(−0.51–1.29)	.393			
Procedure duration	0.00	(−0.03–0.02)	.888			
Study cohort						
Cohort 1	REF					
Cohort 2	−0.72	(−1.75–0.3)	.168	−0.95	(−1.92–0.02)	.055
Cohort 3	−0.62	(−1.57–0.34)	.204	−0.60	(−1.5–0.3)	.192
Cohort 4	−1.69	(−2.66–-0.73)	<.001	−1.40	(−2.31 to −0.48)	**.003**
Access-site bleeding complication	3.23	(2.35–4.11)	<.001	3.18	(2.3–4.07)	**<.001**

*P*-values represent overall comparisons across study cohorts (cohorts 1–4), reflecting temporal changes during protocol implementation. The French size relates to the outer sheath diameter. Abbreviations: BMI = body mass index; CI = confidence interval; Fr = French size; PFA = pulsed field ablation.

## Discussion

In this study, we compared the sequential implementation of different vascular closure strategies after PVI in patients with AF. We demonstrate that the use of the Prostyle™ VCD for sheaths >7 Fr, particularly when combined with a FO8-suture for smaller sheaths (<8 Fr), reduced bleeding complications while enabling earlier mobilization and facilitating SDD at the same time. Our findings highlight the importance of selecting the appropriate vascular closure strategy after catheter ablation for AF.

Although major bleeding complications after PVI are uncommon, minor bleeding complications, including continued venous oozing of the puncture site and groin hematomas, even prolonging hospital stay, are commonly observed. For example, in the study by Lakshmanadoss et al,^6^ no major bleeding events occurred, whereas minor bleeding complications were reported in 18 out of 284 patients. Moreover, Abeln et al,^5^ observed 34 out of 402 patients with minor access-site bleeding complications. Although these minor bleeding complications do not require (invasive) therapeutic interventions, our findings show that they often lead to prolonged bed rest, thereby delaying time to mobilization and discharge. This can have a substantial impact on clinical workflow and healthcare costs, and makes it challenging to pursue SDD for all patients, thereby reducing availability for other patients in a setting with limited healthcare capacity. Although FO8-sutures are relatively inexpensive, these initial cost advantages may be offset by the increased need for care associated with minor bleeding complications. VCDs, although associated with higher upfront costs, may reduce bleeding complications and facilitate earlier mobilization, in combination with an FO8-suture.^23^ Therefore, a combined strategy of VCD and FO8-suture may represent a cost-effective approach by increasing SDD rates and improving overall resource efficiency.

In our study, 2 of 170 patients in cohort 1 (closure with FO8-suture) experienced a major bleeding complication, whereas none of the 568 patients treated after the introduction of the VCD had a major bleeding complication. However, minor bleeding complications were frequently observed, with an incidence of 19.6% in cohort 1, which decreased to 9.6% in cohort 4. Our results show that the change from an FO8-suture protocol (cohort 1) to a VCD protocol (cohort 2 and 3) did not influence the incidence of bleeding complications. This is in line with previous studies that failed to demonstrate a significant difference in bleeding complications between an FO8-suture and a VCD in patients undergoing PVI.^13^^,^^17^ However, the subsequent implementation of a combination protocol of an FO8-suture and VCD (cohort 4) was associated with a marked reduction in bleeding events with an OR of 0.38, despite the shorter bed-rest time. We hypothesize that the change to a VCD alone was not sufficient, because the device was used to close the larger puncture site only, whereas the small puncture site from the diagnostic catheter was not accounted for. The use of an FO8 suture as an adjunct to the VCD allowed the diagnostic-catheter access-site to be closed as well, without the need for long bed rest.

Recently, a meta-analysis^21^ of randomized controlled trials demonstrated lower access-site complication rates with VCD compared with manual compression or FO8-sutures alone. In contrast, our real-world observational data suggest that isolated VCD use remained associated with relatively frequent minor bleeding events, which formed the rationale for implementation of a combined VCD + FO8-suture strategy in cohort 4. Our study additionally reflects routine clinical practice, including mandatory protocol deviations, temporal workflow changes, and operator variability.

In parallel with the sequential implementation of closure protocols, the prescribed ambulation time was reduced from 4 hours of bed rest to 2 hours of bed rest +1 hour seated. In 23.9% of patients in cohort 4, bed rest needed to be prolonged with a median extension of 1.8 hours, mostly due to post-procedural drowsiness following sedation, including both general anesthesia and PSA (42.6%), minor bleeding (38.3%), or logistical reasons. Although 2 hours of bed rest appeared to be insufficient for a subset of patients, this protocol was adequate for three-quarters of the cohort and supported shorter hospital stay. The results showed that the TTD significantly decreased during the evolution of the study cohorts from 7.6 hours to 5.0 hours, because of reduced bleeding complications in combination with the implementation of the shortened bed-rest protocol. In addition, the number of patients discharged on the same day increased from 56.5% in cohort 1 to 67.0% in cohort 4 due to shorter bed-rest times, without negative impact on the proportion of patients who needed an unplanned ONS and/or ER visit because of groin complications, consistent with recent European Heart Rhythm Association consensus statements supporting the safety and feasibility of same-day discharge in selected patients.^24^ The observed reduction in TTD is consistent with a previous study by Fabbricatore et al,^9^ which demonstrated significantly shorter discharge times with VCD use compared with a FO8-suture (FO8-suture: 10:16 ± 1:21 vs VCD: 5:48 ± 1:03 hours, *P* < .001). Similarly, a study by Castro-Urda et al,^25^ comparing VCD combined with an SDD protocol to an FO8-suture strategy with overnight stay, demonstrated a significantly shorter TTD in the VCD group (FO8-suture, 27.09 ± 2.29 vs VCD, 5.89 ± 7.47; *P* < .05), without an increase in emergency department visits.

Our results showed no association between the catheter type (which differs in outer sheath diameter) and bleeding complications, suggesting that vascular closure strategies may play a more important role than sheath sizes in the prevention of bleeding complications. Notably, up to this moment, no other study on the impact of sheath size on access-site bleeding complications has been published.

The very recent ULYSSES randomized controlled trial demonstrated superior safety for ultrasound-guided femoral vein access for AF ablation (1.6% vs 3.3% major AE rate). However, in contrast to previous studies,^26^, ^27^, ^28^ the use of ultrasound guidance was not independently associated with bleeding complications in our cohort. This may be explained by the fact that all operators were very experienced electrophysiologists with a caseload of more than 200 ablation procedures per year. It may also reflect the benefits of proper closure to address post-procedure hemostasis.

### Limitations

This study has several limitations. First, this was a single-center observational study without randomization between vascular closure protocols, introducing the potential for temporal, operator-related, and secular trend confounding. Therefore, although cohort 4 demonstrated lower bleeding rates, it cannot be fully determined whether this was in some way related to the intervention itself or to temporal changes in procedural practice. For example, the use of ultrasound guidance during venous access differed between cohorts due to operator preference and because this became more fashionable in routine clinical practice during the study period.

Furthermore, protamine use increased during the duration of this study, which might have had an influence on bleeding propensity and might have contributed to the positive effect of cohort 4. Therefore, additional analyses, excluding patients receiving protamine, were performed, which demonstrated an association between cohort 4 and lower bleeding rates. However, this was no longer significant, likely due to reduced sample size and low event rates. These findings suggest that increased protamine use may have partially contributed to the observed reduction in bleeding complications across cohorts.

Furthermore, not all bleeding events necessarily reflect direct failure of the venous closure strategy itself. For example, 1 major bleeding event was related to injury of the medial circumflex femoral artery, which likely represents arterial injury rather than venous closure failure.

In addition, a single weight-based bolus of heparin was administered without ACT monitoring; therefore, our findings may not be generalizable to ACT-guided strategies or protocols targeting higher ACT levels without protamine use. Of note, we did not observe any case of transient ischemic attack, stroke, or systemic embolism in our study cohorts.

The use of VCDs may be subject to a learning curve. A previous study^29^ in the arterial access setting has demonstrated that vascular complication rates decreased with increasing operator experience. Although unsuccessful VCD deployment occurred more frequently during earlier cohorts, this was not associated with higher bleeding rates compared with the initial cohort without VCD use. However, increasing operator experience over time may still have partly contributed to the observed outcomes. Finally, deviations from the intended protocol occurred (see Supplemental Appendix 1 for frequency and indications), including crossover to alternative treatments, (particularly in cases of incomplete or unsuccessful VCD deployment requiring adjunctive FO8-sutures), which may have influenced the observed outcomes and introduced bias. Because patients were analyzed according to the initial treatment strategy (intention-to-treat–like approach), these deviations may have attenuated differences between groups. However, this approach reflects real-world clinical practice and supports the generalizability of our findings.

## Conclusion

In conclusion, our results showed that the use of a VCD in combination with an FO8-suture was associated with lower observed rates of access-site bleeding complications and with improved post-procedural workflow of PVI, enabling earlier discharge.

## Disclosures

I.B. is supported by a grant from Boston Scientific. V.D. reports being a consultant for Medtronic and Boston Scientific. M.W. reports being a speaker for Abbott. M.L. reports being a consultant/proctor for Medtronic and Abbott. L.B. reports being a consultant, speaker, and proctor for Boston Scientific, Medtronic, and ArgaMedTech. All fees go to the cardiology department. The remaining authors declare no conflicts of interest.
