# Supplementary material for: Vascular closure strategies in pulmonary vein isolation to reduce vascular complications and facilitate same-day discharge
Source: Heart Rhythm O2. 2026 Jul 13;7(10):1856–65. doi: 10.1016/j.hroo.2026.07.005 (PMC13700363; doi:10.1016/j.hroo.2026.07.005)
Supplement: Supplementary file 1 [file mmc1.docx]

**Appendix 1**

*Protocol deviations:*

The following deviations from the prespecified access-site vascular closure protocol were observed across study cohorts:

In cohort 1 (FO8-suture + 4h bedrest), one/170 patient required two VCDs because an unintended arterial puncture had occurred.

In cohort 2 (VCD + 4h bedrest), 13/161 patients received an additional FO8-suture due to leakage after VCD deployment. In five patients, VCD deployment was unsuccessful, and a FO8-suture was applied. One patient required two VCDs because of persistent oozing after the first device.

In cohort 3 (VCD + 2h bedrest + 2h seated), VCD deployment was (partially) unsuccessful in 20/ 210 patients. Eighteen of these patients subsequently received a FO8, while two patients were managed with an additional two hours of bedrest.

In cohort 3/197 (VCD + 2h bedrest + 1h seated), VCD deployment was unsuccessful in three patients, who were treated with an additional two hours of bedrest. In addition, three patients required two VCDs to achieve hemostasis.
